# Hyperspectral abdominal laparoscopy with real-time quantitative tissue oxygenation imaging: a live porcine study

**DOI:** 10.3389/fmedt.2025.1549245

**Published:** 2025-06-05

**Authors:** Oscar MacCormac, Conor C. Horgan, Dale Waterhouse, Philip Noonan, Mirek Janatka, Richard Miles, Jaco Jacobs, Cameron Dockerill, Théo Trotouin, Alexis Schizas, Barbara Seeliger, Sebastien Ourselin, Michael Ebner, Tom Vercauteren, Jonathan Shapey

**Affiliations:** ^1^ School of Biomedical Engineering & Imaging Science, King’s College London, London, United Kingdom; ^2^ Department of Neurosurgery, King’s College Hospital, London, United Kingdom; ^3^ Hypervision Surgical Limited, London, United Kingdom; ^4^ Department of General Surgery, St Thomas’ Hospital, London, United Kingdom; ^5^ Institute of Image-Guided Surgery, IHU Strasbourg, Strasbourg, France; ^6^ Department of Digestive and Endocrine Surgery, University Hospitals of Strasbourg, Strasbourg, France; ^7^ ICube, UMR 7357, CNRS, INSERM U1328 RODIN, University of Strasbourg, Strasbourg, France

**Keywords:** hyperspectral imaging, laparoscopy, minimally invasive surgery, tissue oxygenation, StO_2_, anastomosis

## Abstract

**Background:**

Ischaemia is a critical complication, and can result in poor surgical outcomes. While intra-operative overt ischaemia can be perceived with the naked eye, timely recognition of borderline perfusion can prevent post-operative ischaemic complications, which is particularly relevant for colorectal anastomoses. Consequently, there is a clinical need for new technologies to intra-operatively assess tissue oxygenation (indicative of end organ perfusion), with minimal disruption to the surgical workflow. Here we present a hyperspectral imaging (HSI) system for laparoscopic surgery. This system provides live, easy to interpret, tissue oxygenation (StO_2_) maps with associated quantitative values.

**Methods:**

White light view and tissue oxygenation maps were reconstructed from a protoype laparoscopic Hyperspectral Surgical System (HSS). First, in a live porcine model (55 kg female), the mesentery of a small bowel loop was temporarily occluded with a laparoscopic grasper, then released whilst being imaged with HSI. The quantitative StO_2_ values obtained from the HSS were compared with those of a non-invasive tissue oximetry probe (Moor VMS-Oxy, Moor Instruments Ltd, United Kingdom). Secondly, mimicking a laparoscopic colon resection and anastomosis, the colorectal junction was mobilised laparoscopically, exteriorised, transected, anastomosed and repositioned in the abdominal cavity. In order to compare healthy and ischaemic colon, the distal part was intentionally devascularised. Tissue oxygenation maps were compared with indocyanine green fluorescence angiography (ICG-FA) of the anastomotic region.

**Results:**

The HSS was used as the primary scope to complete a laparoscopic colorectal anastomosis, providing a simultaneous white light view and hyperspectral information. Quantitative results from small bowel imaging were shown to correlate with measurements from the superficial tissue oximetry probe. Real-time tissue oxygenation maps were shown to visually correlate with ICG-FA.

**Conclusion:**

The HSS can guide laparoscopic surgical procedures whilst providing visual and quantitative tissue oxygenation information in a live animal model. This paves the way for further studies to assess clinical applications.

## 1 Introduction

Laparoscopic surgery has long proven to be safe and cost effective, resulting in reduced recovery times when compared to open surgery (1, 2). For optimal surgical vision, there has been continuous improvement in the associated visual technology over the last three decades, now with ultra-HD/4K and 3D laparoscopy systems being used (3). Furthermore, these improved imaging techniques have been associated with improved surgical outcomes (4, 5). Despite these significant improvements in standard white light imaging on conventional red, green and blue (RGB) camera systems, there remain invisible tissue parameters that may have significant impact on the success of the surgery. One such parameter is tissue oxygenation (StO_2_), and its impairment has been demonstrated to be an important factor in the aetiology of surgical complications, including anastomotic leakage (AL) (6).

Bowel anastomoses are a common procedure, particularly following oncological abdominal surgery (6), with nearly 85% of colorectal resections resulting in primary anastomosis in the UK (7). Whilst AL incidence is usually as low as 6%–7%, it can be as high as 19% in colorectal anastomoses (6, 8–12). When AL does occur, it carries a significant morbidity of up to 98%, with a long-term mortality as high as 36.4% (11, 13, 14). Moreover, it is associated with a significantly increased local tumour recurrence in colorectal cancer resections (9, 15–18).

For uncomplicated healing of a bowel anastomosis, adequate blood supply is considered essential to avoid AL (8, 13, 14, 19–21). Appropriate delivery of nutrients, in particular oxygen (22, 23), is essential for cellular homeostasis, function and wound healing (24). In ambient air, oxygen is delivered to tissue via blood, principally (98%) reversibly bound to haemoglobin in red blood cells (measured as oxygen saturation), and only a little dissolved in blood plasma. However, oxygen delivery also depends on other factors i.e., increased temperature, decreased pH and increased carbon dioxide (${\mathrm{CO}}_{2}$) facilitate offloading of oxygen from haemoglobin. This is referred to as a ‘right shift’ in the oxygen dissociation curve (25). Aside from such general and systemic factors, it is generally assumed that a tissue that is well perfused receives an adequate oxygen supply. Detailed assessment of perfusion via blood flow or tissue oxygenation measurements could provide complementary information, particularly in situations of hypoxia or reduced oxygen carrying capability (low haemoglobin) (26).

Unfortunately, visualising bowel perfusion and oxygenation remains a challenge to predict anastomotic blood supply or tissue oxygenation (27). A number of approaches have been proposed to improve perfusion assessment with associated evidence to demonstrate improved patient outcomes, including indocyanine green fluorescence angiography (ICG-FA), laser speckle contrast imaging (LSCI) and Hyperspectral Imaging (HSI) (14).

Indocyanine green (ICG) fluorescence angiography (ICG-FA) is the method described most in the literature (14) and is likely the most familiar amongst general surgeons (28, 29). ICG is a fluorophore with an excitation peak within the near infra-red (NIR) spectrum (∼780 nm) and has an excellent safety profile (30, 31). It can be injected systemically into the bloodstream, or locally delivered via the urethra or submucosally depending on the organ/tissue of interest to be visualised (32).

Laser speckle contrast imaging (LSCI) is a wide-field technique for visualising microvascular perfusion in near real-time (33, 34). LSCI has only recently been integrated into a minimally invasive (laparoscopic) surgical setup (35, 36).

Unfortunately, neither ICG-FA nor LSCI are able to directly quantify StO_2_ information with currently commercialised systems, but rely on measuring tissue perfusion, from which oxygen supply may be inferred.

**Figure 1:**
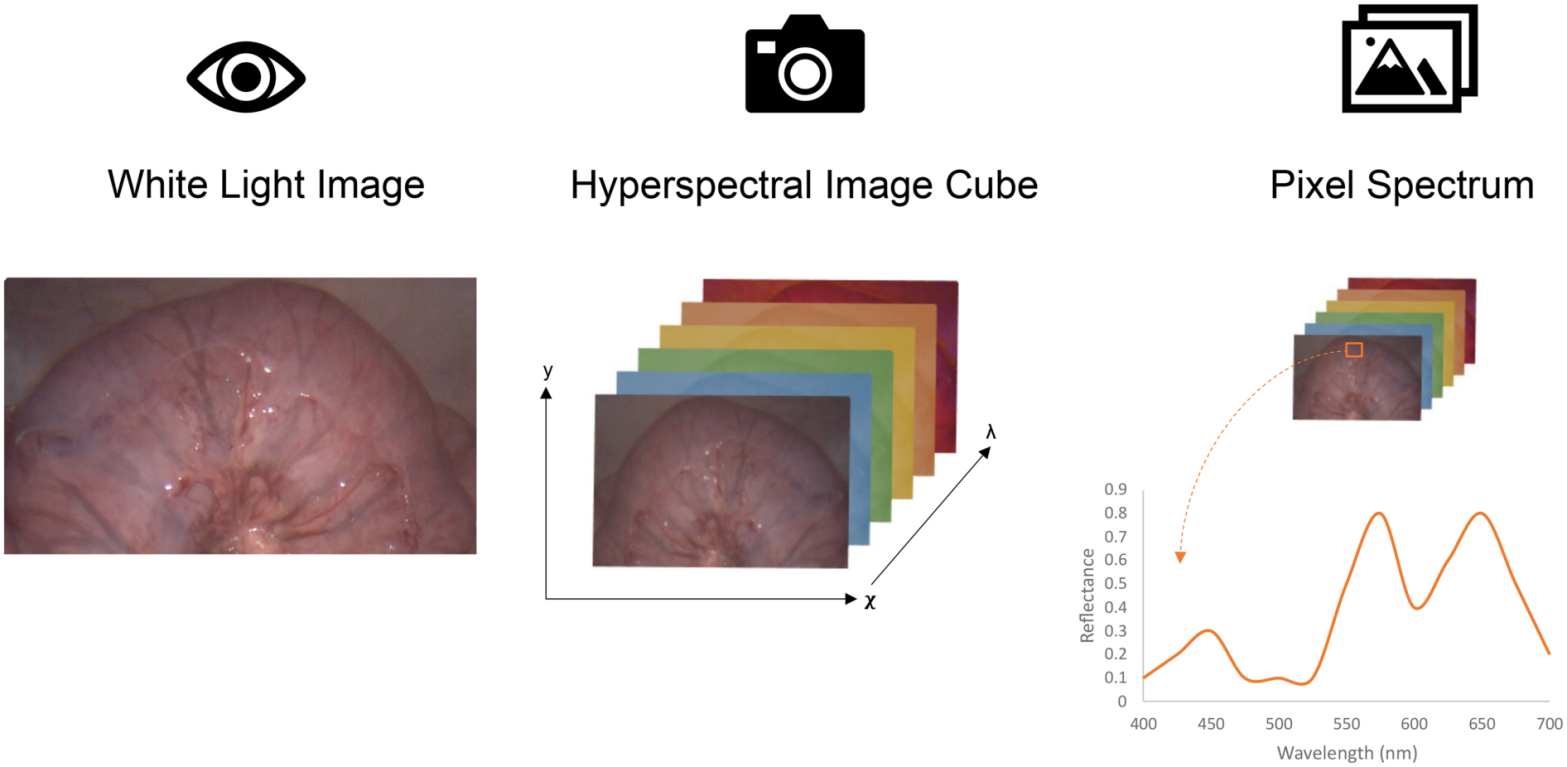
(Left) Simple 2D white light image. (Middle) 3D (two spatial: χ,γ and one spectral: λ) HSI image, resulting in a complete hypercube. (Right) Schematic representation of a HSI generated spectral curve from defined region of interest.

Hyperspectral imaging (HSI) is a wide-field, spectral imaging technique that is capable of obtaining specific tissue information depending on the optical properties of the structure in question, especially its scattering and absorption (37). By returning spatially-resolved, multi-channel spectral information, where each channel corresponds to a narrow wavelength band, each pixel in the image will have its own *spectral signature* as illustrated in Figure 1. Snapshot hyperspectral imaging captures the entire spatio-spectral data cube in a single image, meaning it can image in real-time, albeit with some compromises to spatial and spectral resolution, making it particularly suitable for clinical applications where live, real-time imaging is essential (38).

More specifically, snapshot mosaic HSI is a camera-based method by which each pixel has a single optical band-pass filter, allowing collection of a single channel per spatial location in one image (39, 40). Spectral filters are arranged in a repeating mosaic fashion, usually 4 × 4 or 5 × 5 pixels resulting in a lower spatial resolution per spectral channel and misalignment across spectral channels (39). Post-processing steps can infer the complete spatio-spectral data from the sampled pixels, improving the overall spectral and spatial resolution (41, 42). By simultaneously combining snapshot mosaic HSI with computational post-processing steps, it has been demonstrated as a feasible, real-time method for providing tissue differentiation and oxygenation measurement capabilities in surgery (43–47).

Hyperspectral imaging offers the potential for label-free, real-time, high-resolution imaging and tissue oxygenation information intra-operatively without many of the limitations described for ICG-FA (48–51). However, much of the current clinical literature pertaining to intra-operative HSI systems has so far been limited to open surgical techniques (52). This is due to the size of previous HSI cameras, as well as slow acquisition times, making these systems generally unsuitable for laparoscopic/minimally invasive surgery (MIS) (53). Diaspective Vision have made way towards overcoming these initial obstacles by developing an intra-operative laparoscopic HSI system (TIVITA Mini) that has demonstrated feasibility for *in vivo* use (53–55). Ayala et al. (43) have also taken promising steps towards addressing some of the challenges associated with intra-operative MIS HSI and have published the only clinical study in the literature reporting a laparoscopic HSI system capable of acquiring real-time tissue perfusion information, albeit in conjunction with a standard white light laparoscope (43). However, neither of these systems are yet able to provide truly quantitative and real-time StO_2_ information and simultaneously be used to guide entire laparoscopic procedures.

Further developments have been made to improve upon these promising starts towards hyperspectral-guided laparoscopic surgery (41, 45). An early prototype laparoscopic hyperspectral surgical system (HSS), provided by Hypervision Surgical Ltd, utilises a snapshot mosaic camera integrated with a standard laparoscopic setup and is able to provide real-time, high resolution (1,088 pixels × 2,043 pixels), hyperspectral generated RGB video as well as simultaneous real-time tissue oxygenation (StO_2_) information.

The aims of this proof of concept study are to assess the reliability and feasibility of the HSS to guide laparoscopic abdominal surgery by performing a laparoscopic rectal mobilisation with externalised anastomosis, compare real-time StO_2_ information with the current clinical standard (ICG-FA), demonstrate the HSS capability to provide quantitative StO_2_ information by inducing small bowel ischaemia in a live porcine model and obtain initial surgeon feedback regarding the usability of the system.

## 2 Materials and methods

### 2.1 Equipment used

To enable simultaneous real-time white light imaging and quantitative imaging of superficial tissue oxygenation, this study used a prototype laparoscopic hyperspectral imaging system (Hypervision Surgical Ltd) with manual focus and fixed magnification. The sensor is a 4×4 snapshot mosaic sensor with wavelengths covering the visible range. This system provides simultaneous real-time (60 fps) acquisition and processing of white light images alongside superficial tissue oxygenation visualisation based on computational hyperspectral imaging using proprietary artificial intelligence (AI) driven algorithms. A standard sterile camera drape was used to cover the HSS camera head and was coupled with a standard sterile laparoscopic telescope (Hopkins $0^{\circ }$ laparoscope, Karl Storz, Germany). This ensured sterility of the overall imaging system as per standard clinical practice. The display, Sunoptic Titan X450 Xenon light source (Sunoptic Technologies, Jacksonville, USA) and computer system were mounted on a standard medical cart to allow bedside positioning of the system. For ICG imaging, an independent commercially-available ICG imaging system (EleVision IR platform, Medtronic, USA) was deployed alongside the HSS.

### 2.2 Experimental animal procedure

**Figure 2:**
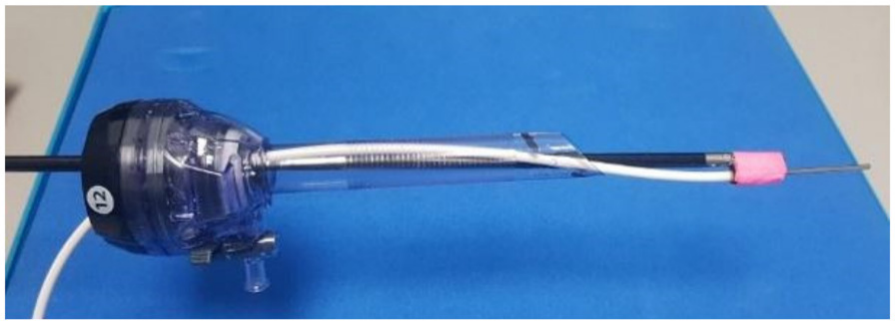
Tissue oximetry probe secured to a Johan laparoscopic grasper and passed via a 12 mm laparoscopic port.

On 12th June 2023, a female 55 kg Landrace pig was anaesthetised using Zoletil™ 2.2 mg/kg and medetomidine 0.02 mg/kg administered intramuscularly. General anaesthesia was induced with oxygen over isoflurane via a close fitting face mask, before intubation with a cuffed endotracheal tube (ETT). Anaesthesia was then maintained with oxygen over isoflurane via the ETT and respiration controlled via a ventilator. The animal was then positioned supine and the ambient lighting was switched off whilst the HSS was white balanced. A small (15 mm) midline incision was made and a 12 mm trocar placed under direct vision before peritoneal insufflation with CO_2_ to a pressure of 12 mm Hg. The laparoscopic HSS was inserted via this port and two further 12 mm ports along with one 5 mm port were placed under direct vision, using the real-time RGB video display from HSS, as per standard laparoscopic surgery procedure. Inside the abdomen, the surgeon selected a suitable small bowel loop and its corresponding mesentery was occluded with a laparoscopic grasper. Raw HSI data was recorded throughout the procedure, and simultaneous RGB and StO_2_ information was reconstructed for display to guide the procedure. The grasper was then released and reperfusion imaged and recorded using the HSS. A point-based superficial tissue oximetry probe (Moor VMS-Oxy, Moor Instruments Ltd, United Kingdom) was used to obtain quantitative StO_2_ measurements from relevant tissues during the procedure, based on the work published by Saito and Yamaguchi (56). The tissue oximetry probe was introduced into the abdomen alongside a standard laparoscopic grasper via a 12 mm port (Figure 2) and manipulated with the grasper.

Once the tissue oximetry probe was positioned onto the same selected small bowel loop, the HSS light source was switched off to ensure accurate readings from the tissue oximetry probe. The tissue oximetry probe was held steady and StO_2_ readings were recorded (phase i). The HSS light source was then switched back on and the mesentery was temporarily occluded under direct vision, (phase ii). The HSS light source was switched off once again with the tissue oximetry probe held in the same position. StO_2_ readings were recorded until no further oxygenation decrease was observed (phase iii). The HSS light source was then switched on, with RGB visualisation of the ischaemic segment, and the mesentery released (phase iv), with the tissue oximetry probe then being positioned again and the HSS light source switched off. The tissue oximetry probe recorded StO_2_ measurements following reperfusion (phase v).

This process was repeated using the HSS. However, phase ii and iv were not required, given that recording of well perfused tissue (phase i), mesentery occlusion and ischaemia (phase iii) and reperfusion/return to normal values following mesentery release (phase v) could be carried out entirely under the visual guidance of the continuous wide field imaging of HSS. This process is summarised in Table 1.

**Table 1:** Table outlining the procedure for tissue oximetry probe recordings and HSS recordings before, during and after induced small bowel ischaemia.

Point based StO_2_ measurement using the tissue oximetry probe			
Phase	HSI system light source	Tissue oximetry probe	Procedure
(i)	OFF	Recording	Tissue oximetry probe held perpendicular to selected small bowel loop.
(ii)	ON	Not recording	Small bowel loop mesentery identified and occluded.
(iii)	OFF	Recording	Tissue oximetry probe held perpendicular to selected small bowel loop.
(iv)	ON	Not recording	Small bowel mesentery released, permitting re-perfusion of small bowel loop.
(v)	OFF	Recording	Tissue oximetry probe held perpendicular to selected small bowel loop.
Wide field continuous StO_2_ visualisation using the HSS			
Phase	HSI system light source	Procedure	
(i)	ON	Selected small bowel loop imaged using HSS.	
(iii)	ON	Small bowel loop mesentery identified and occluded. Induced ischaemia imaged using HSS.	
(v)	ON	Small bowel mesentery released and reperfusion imaged with HSS.	

In total, two epsiodes of ischaemia were induced for the same segment of small bowel, one with the HSS light source predominantly off (tissue oximetry probe recording) and one with the HSS light source predominantly on (HSS recording).

Following this, a HSI laparoscope guided colorectal anastomosis was performed. The colorectal junction was mobilised laparoscopically and subsequently exteriorised via a mini laparotomy. The colon was transected and a continuity re-established with a hand sewn anastomosis prior to returning the colon to to the abdominal cavity. StO_2_ was not monitored during the exteriorised, hand-sewn anastomosis creation. The mini laparotomy was closed with silk sutures to maintain peritoneal insufflation thereafter. Subsequently, the porcine cranial rectal artery, which is equivalent to the human superior rectal artery (57), was occluded using Ethicon Ligaclips under HSI laparoscopic guidance and the marginal vasculature divided along the rectum, with StO_2_ information displayed and recorded using the HSS. This resulted in a near total devascularisation of the rectum, which facilitated a clear delineation between well perfused colon proximal and ischaemic distal to the anastomosis.

In order to create a comparison with ICG, a NIR compatible laparoscope (EleVision system) was used via a port adjacent to the HSS. ICG solution was prepared and injected intravenously at a dose of 0.2 mg/kg and the NIR system laser switched on, whilst the HSS light source was switched off. ICG-FA video was recorded up to to 5 min following ICG injection.

#### 2.2.1 Comparison with ICG

Once the colorectal junction was mobilised laparoscopically, exteriorised, transected, anastomosed and repositioned in the abdominal cavity, the HSS was used to visualise StO_2_ in the well perfused tissue proximal to the anastomosis and the poorly perfused tissue distal to the anastomosis. Following injection of ICG, the Medtronic EleVision system was used to visualise ICG fluorescence in the same regions. Recordings were taken from each system, and a direct visual comparison was made.

#### 2.2.2 HSI guided laparoscopic surgery

The surgical procedure was conducted by a qualified general surgeon with vast experience in laparoscopic surgery. Following the procedure, a short interview was conducted with the surgeon. He was asked to comment on the image quality, colour matching and usability of the HSS compared to the current clinical standard for laparoscopic imaging. Since a single surgeon operated on a single animal, no formal analysis of the surgeon feedback was performed. The interview was taken as an initial indication of system potential and usability by a single end user.

Following the experimental procedure, the pig was immediately euthanised with intravenous sodium pentobarbitone 140 mg/kg, in accordance with UK schedule 1 methodology.

IRB approval and written consent were not required for this study, as this study did not involve human participants.

## 3 Results

The HSS was used to successfully complete the experimental procedure as planned. There were no procedure related complications during this study, with no bleeding or initial indicators of anastomotic leakage.

### 3.1 ${\mathrm{StO}}_{2}$ visualisation in small bowel

**Figure 3:**
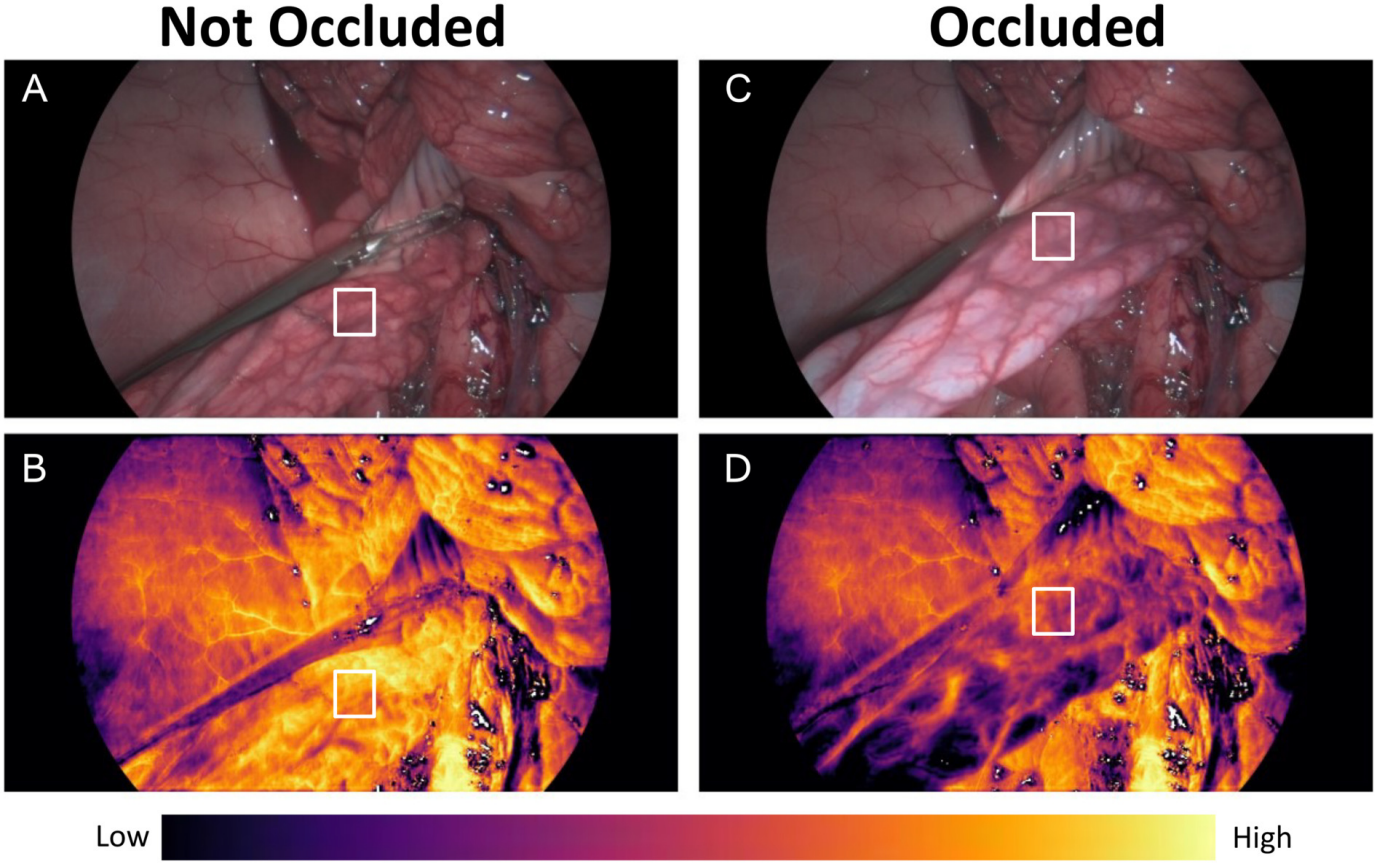
**(A)** Small bowel with open grasper and mesentery not occluded in RGB. **(B)** Small bowel with mesentery not occluded in StO_2_ vision. **(C)** Small bowel with closed grasper and mesentery occluded in RGB. **(D)** Small bowel with mesentery occluded in StO2 vision. Tissue oxygenation map colour bar denotes areas corresponding to low and high StO_2_. White square denotes the region at which the tissue oximetry probe was placed to generate the graph seen in Figure 4.

Under laparoscopic vision with the HSS, hyperspectral-based reconstruction of RGB video was used to guide identification and occlusion of a small bowel mesentery. Figures 3A, C demonstrates the hyperspectral-based reconstruction of RGB video that was used to guide identification and occlusion of a small bowel mesentery. Whilst slight colour changes may be appreciated in the RGB images following occlusion of the mesentery, the HSS generated StO_2_ colour map demonstrated a more distinct and objective visualisation of the reduction in bowel StO_2_ (Figures 3B, D).

**Figure 4:**
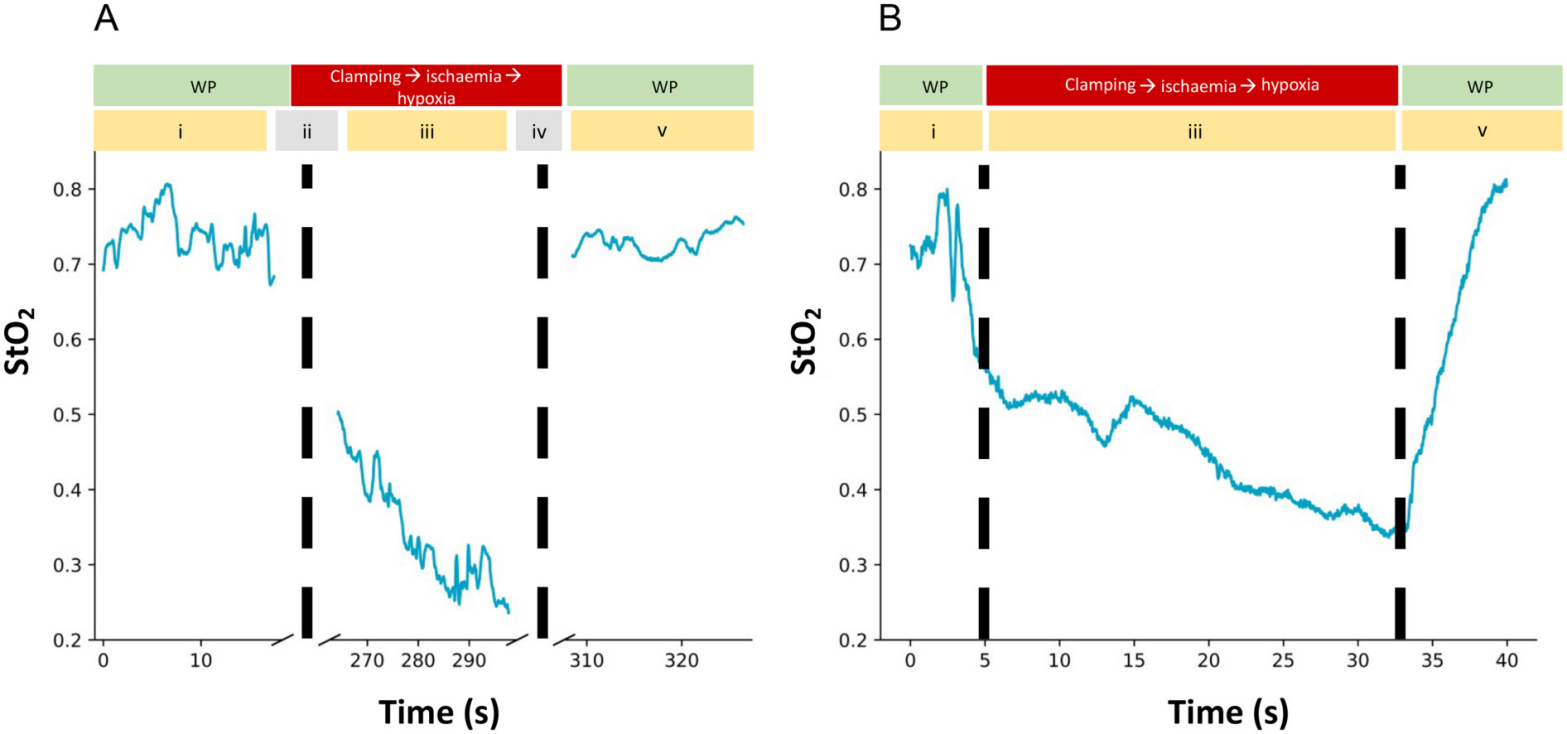
**(A)** tissue oximetry probe readings from well perfused (WP) small bowel (phase i), during induced ischaemia and reperfusion (phase iii) and following reperfusion (phase v). Dotted lines represent the clamping (phase ii) and release (phase iv) of the small bowel mesentery, during which no StO_2_ data could be recorded because the HSS light source was switched on for visualisation of the grasper. **(B)** HSS generated quantitative StO_2_ readings from well perfused (WP) small bowel (phase i), during induced ischaemia (phase iii) and reperfusion of the small bowel (phase v). Dotted lines represent the time points where the small bowel mesentery was occluded and released.

The region of interest (RoI) highlighted by the white rectangle in Figure 3 represents the tissue oximetry probe measurement area, providing reference readings for bowel StO_2_ during ischaemia and reperfusion as shown in Figure 4.

### 3.2 Comparison with ICG

**Figure 5:**
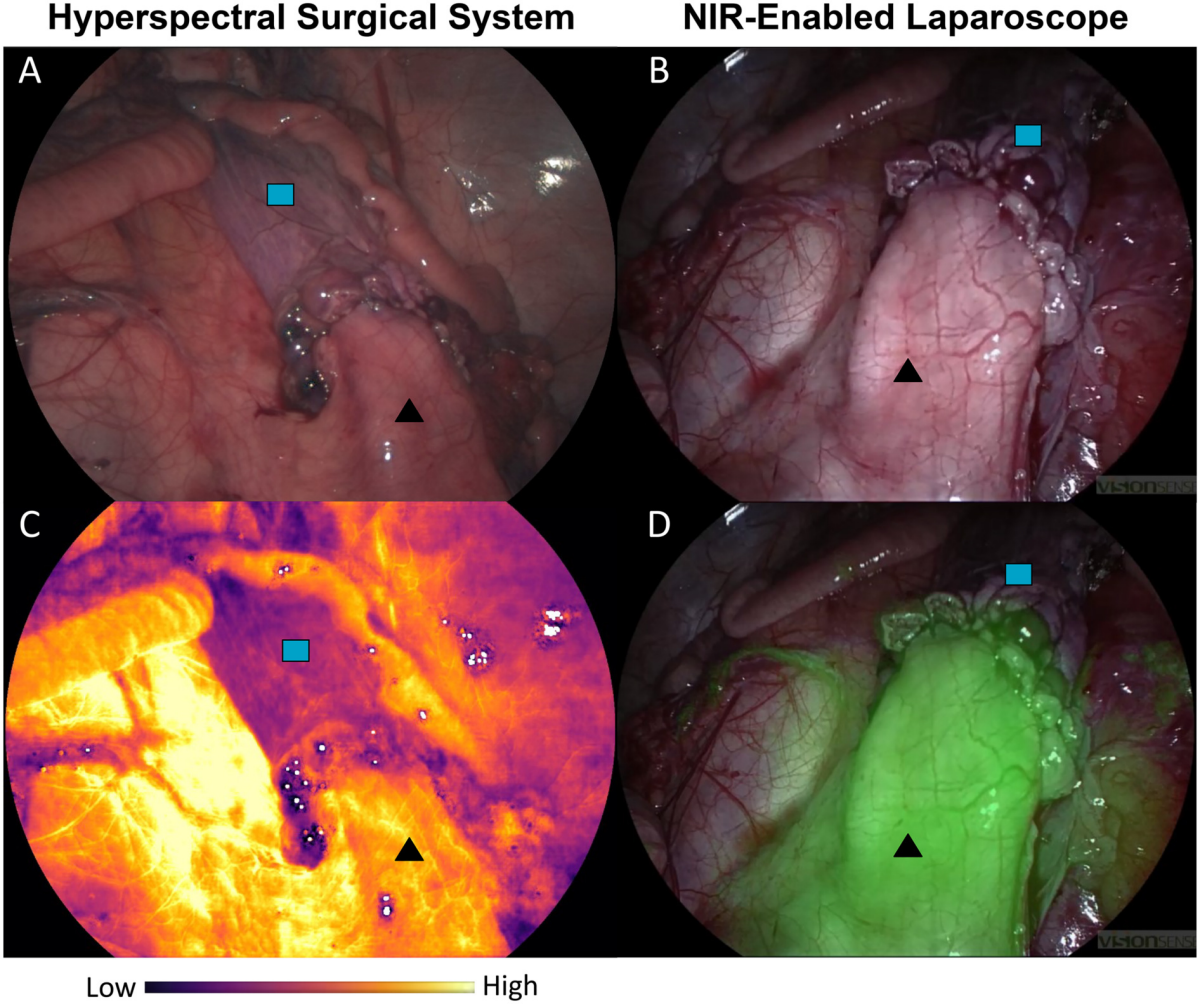
Comparison of ischaemic 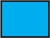 rectum and well perfused 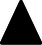
colon. **(A)** HSI-generated RGB. **(B)** Near-infrared (NIR) enabled laparoscope RGB. **(C)** HSI-generated StO_2_ map. **(D)** NIR overlay of the same anastomotic region. Different viewpoints of the anastomosis are due to imaging via different ports to accommodate both the HSS and laparoscope (Ele Vision).

Following completion of the colon anastomosis, with intentional devascularisation of the distal colon/rectum, the HSS was capable of providing StO_2_ information for the deoxygenated rectal tissue distal to the anastomosis as shown in Figure 5.

Whilst colour differences can be perceived in the HSI-generated RGB in Figures 5A, B, the visual (non-quantitative) assessment of the ICG-FA view in Figure 5D also demonstrates the ischaemic boundary. The HSI-based StO_2_ map illustrated the well oxygenated and deoxygenated regions comparatively clearly in Figure 5C.

### 3.3 HSI-guided laparoscopic surgery

**Figure 6:**
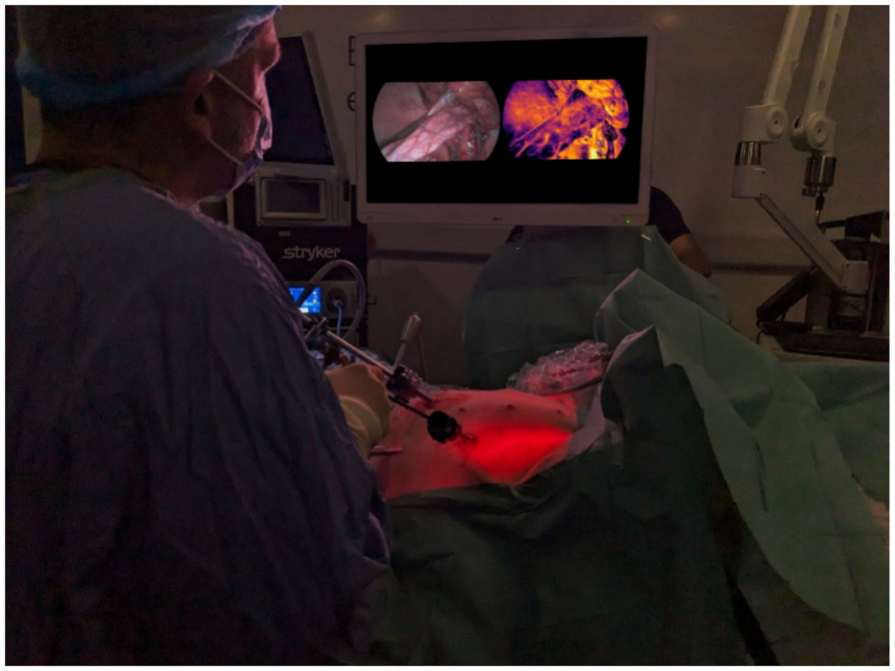
Use of the HSS for laparoscopic surgery. It simultaneously provides the RGB view generated from hyperspectral information (left on the display monitor) as well as the StO_2_ colour map display (right on the display monitor). The image displayed on the screen has been replaced to better represent the visualised StO_2_ map.

The HSS was successfully used to carry out laparoscopic surgery as it displayed real-time HSI-generated RGB images (akin to standard white light imaging), whilst simultaneously displaying real-time StO_2_ information next to the RGB feed as illustrated in Figure 6.

During the post-procedure interview with the lead surgeon, points highlighted were that the system was “easy to use, handle and not heavy,” as well as “The frame rate is good without perceptible lag.” Additionally, the operating surgeon felt that “the colours with the HSS RGB seem more accurate than that of the laparoscopic system,” referring to the ICG imaging system used in this study. Areas to improve in the pre-commercial HSS used in this early study were that “the [image] definition is closer to standard definition than high definition” and that “the [image] definition is not as good when viewing at a distance.” These have been elaborated on further in Section 6.

## 4 Study limitations

Whilst we feel that this study shows promising early results, it is important to emphasise its limitations. Firstly, this is a proof of concept using a single animal, with a single independent operating surgeon. The qualitative feedback was also from this one surgeon. Formal qualitative assessments would need to be carried out to thoroughly assess this technology, its usability and its impact on the wider operating theatre team, akin to those performed with other, similar hyperspectral systems (58).

Additionally, whilst every effort was made to create the same surgical environment when data was being collected using the point based tissue oximetry probe and the HSS, the steps outlined in Table 1 meant that the time points for each method were not the same, as evidenced in Figure 4. This means that a meaningful statistical analysis was not able to be performed on the single set of results obtained and further studies would need to be carried out to facilitate a detailed statistical analysis.

A further limitation of this proof-of-concept study is that it was designed to compare StO_2_ visualisation of ischaemic and oxygenated bowel following the anastomosis. Further studies will be required to assess the system’s ability to detect more subtle and transitional ischaemic changes, such as those observed in the ischaemia-reperfusion StO_2_ curves, as well as to inform clinical decision making and assess the impact of such image guidance on the clinical outcome.

## 5 Barriers to uptake of new surgical technology

When considering developing a new technology, it is important to consider perceived barriers to adoption, which have been well described. From an individual perspective, how useful the technology is and how well it integrates into an individual surgeon’s practice has been shown to be a key factor (59, 60), as opposed to how easy the technology is to use, which plays less of a role (60). As well as this, lengthy training time has been demonstrated to reduce uptake of new surgical technologies (59), which we feel is unlikely to play a significant role with the HSS given the fact that our system uses already familiar laparoscopes. Finally, lack of clear benefits to patients is a significant barrier to uptake, which is why further work is required to establish the tangible impact of the HSS on our patient population. We are addressing this by undertaking a multi-centre trial in human participants (LapHSI Clinical Trial Number NCT06700317), due to begin recruitment in 2025.

## 6 Discussion

Our study aimed to demonstrate the reliability and feasibility of a novel HSI system that can provide real-time quantitative StO_2_ information in bowel tissue alongside conventional RGB vision for surgical guidance. This system is also the first system that we are aware of that utilises HSI-generated RGB to guide the entire surgical procedure, without reliance on an additional RGB imaging system.

This study has demonstrated that the HSS is capable of laparoscopic surgical guidance, as well as displaying accurate bowel StO_2_ information to the surgeon in real-time. The HSS HSI generated RGB visualisation was able to guide the surgery throughout the procedure. HSI-generated quantitative StO_2_ results showed a good correlation with readings taken using the tissue oximetry probe, although it is important to note that the probe was used outside of its intended use (FDA cleared for *non-invasive* use). Similarly, the visual StO_2_ map was comparable to the ICG-FA perfusion display, as can be seen in Figure 5.

In terms of limitations reported by the lead surgeon, image definition is an important factor to consider for improvement, with the goal of full HD (1080 × 1920) visualisation desired as a minimum for surgery. From the computational perspective, image resolution improvements are expected by deploying dedicated super-resolution methods. From the hardware perspective, further improvements could also be achieved by using an improved sensor with a greater pixel density.

Related to the above, some feedback from the surgeon detailed in Section 3 suggested that imaging from a greater working distance resulted in slightly poorer image definition. This may be a limitation of the system’s hardware, for example the optical quality of the camera coupler. Similarly, the current HSS utilises a manually adjustable focus that some surgeons may be unaccustomed to. Whilst the system has a large depth of field, focus may need to be adjusted manually to achieve in-focus images at different working distances. Our surgeon typically operates using state-of-the-art 4 K robotic systems with in-built autofocus (Da Vinci Xi); systems typically present in world-class teaching and research hospitals. However, many centers that offer laparoscopic surgery still use standard definition (SD) cameras with manual focus as standard of care. Moreover, the image quality of future systems may be improved by enabling a greater depth of field and integrating an autofocus capability (61, 62), in line with recent MIS system developments.

However, the HSS demonstrates a promising advancement in the field of laparoscopic HSI-enabled surgical vision, in line with the progress made by Diaspective Vision. Their MIS HSI system (TIVITA-Mini) has been compared with their open surgical system, which has been shown to accurately detect bowel ischaemia, surpassing quantitative ICG-FA in predicting local capillary lactate levels as a marker of ischaemia (49, 63). Correlation has been shown between intra-operative StO_2_ measurements taken using the TIVITA Mini and the open TIVITA system, albeit with a mean absolute error (MAE) between 12.6% and 17.7% (53). However, it is important to note that this correlation was achieved during open surgery, and the response of the system when used as intended inside the abdominal cavity may be different to the response measured outside of it. For example, the presence of multiple distinct tissue types within the same field of view, as well as the enclosure of the system in a semi-opaque cavity will almost certainly affect the optical properties of the system by introducing differences in light scattering, absorption and reflectance when compared to the open surgical environment. Another important limitation with the TIVITA Mini is the fact that a laparoscopic holder was required for stabilization as the handheld system created too much motion blur (53). Whilst using a laparoscope holder is technically feasible in MIS surgery, it might significantly impact the surgical workflow when the system is used throughout multi-quadrant procedures. Furthermore, the reported HSI image acquisition time for the TIVITA Mini was between 4.6 s (54) and 7 s (53), which complicates a seamless integration into the surgical workflow.

Ayala et al. (43) have reported a clinical study in which a laparoscopic multispectral imaging (MSI)/HSI system capable of providing real-time StO_2_ information has been evaluated. However, in their work, they relied on a proposed *ischaemic index* as opposed to an absolute StO_2_ value. This means the results are somewhat binary as the tissue is considered either perfused or ischaemic. Whilst certainly beneficial in the context of establishing whether renal blood flow has confidently been occluded, this may not be as helpful in other surgical applications, such as bowel anastomoses, where the StO_2_ changes can be more subtle. Furthermore, the study required an additional standard laparoscopic camera to carry out the surgery, meaning multiple camera removal and insertions as well as frequent alterations to the laparoscopic light source, resulting in a negative impact on the surgical workflow

Whilst our study focuses on HSI to guide surgery and its ability to provide StO_2_ information, it should be noted that concurrent approaches are being pursued for obtaining tissue perfusion information in laparoscopic surgery. However, as per Section 1, both ICG-FA and LSCI come with their own limitations.

In the context of digestive anastomoses, ICG requires preparation by an anaesthetist and is generally administered as an intravenous bolus when requested by the surgeon (64). However, the protocols worldwide are not fully standardised and assessment is time-dependent (14). Colonic fluorescence is generally monitored for two minutes following injection with the laparoscopic NIR camera/mode. This can also be performed in open surgery with a dedicated camera system provided ambient lighting is switched off (64). There are also further limitations to ICG image analysis. Firstly, it remains subjective. The operating surgeon will need to decide whether the fluorescence visualised is representative of adequate blood supply or (borderline) ischaemia and this is often unclear (16, 65). Whilst steps have been made in correlating certain parameters of the ICG intensity curve, such as maximum fluorescence ($F_{\max}$) and the time to maximum fluorescence ($T_{\max}$), with AL (16, 66–71), there have been inconsistent results in studies thus far (16). Attempts have also been made to use computer software to quantify fluorescence dynamics (51, 65, 72–75), although these remain in the development phase and are not yet available for clinical use outside of clinical study protocols. Repeated doses are often required throughout a surgical procedure to confirm adequate perfusion (76), but background fluorescence from prior injections can hinder visual assessment and poses further challenges for potential quantitative evaluation (77). Conditional factors such as distance of the camera from region of interest (RoI), ambient lighting conditions (64) and patient specific factors such as hypertension/portal hypertension (65) can also affect the fluorescence intensity and time to (peak) fluorescence, making this technique challenging to standardise. Indeed, the ICG-FA shown in Figure 5C could be biased by highlighting the region closest to the camera in green, with a potentially weaker signal imperceptible in the overlay for the distal rectum.

LSCI has only recently been integrated into a minimally invasive (laparoscopic) surgical setup (35, 36, 78). LSCI achieves tissue perfusion monitoring by measuring the interference pattern caused by scattered light from a tissue surface that has been illuminated by a laser (34, 79); the so called “speckle” pattern. When there is motion within the illuminated surface, particularly at a rate equal to or greater than the image exposure time (e.g., blood flow) then this speckle pattern will become blurred (34). This blurring, or contrast loss, can be correlated with tissue perfusion (34, 36). As with ICG-FA, there are also limitations with LSCI. For example, as LSCI relies on the movement of blood, it is also susceptible to other forms of motion such as breathing or peristalsis (80) and is bound to qualitative measurements with very limited inter-patient comparability (34). LSCI is also susceptible to specular reflection and differences in camera angulation, making it subject to inconsistencies when used in the abdominal cavity where surfaces are curved and often reflective (14, 65, 80, 81).

## 7 Conclusion

This presented prototype HSS has enabled the first fully HSI-driven laparoscopic surgery. It is capable of simultaneously providing standard surgical visualisation and simultaneously displaying tissue StO_2_ information to the surgeon. We have shown that the HSS HSI-derived StO_2_ values align well with reference values recorded with a tissue oximetry probe and that it compares favourably with ICG-FA within the confines of this study. This paves the way for further development and future work to demonstrate the utility of the HSS in patients undergoing laparoscopic bowel surgery is planned.

## Data Availability

The original contributions presented in the study are included in the article/Supplementary Material, further inquiries can be directed to the corresponding author/s.
